# The Genus *Ephedra* (Ephedraceae, Gnetales) in Tunisia: New Records with Taxonomic, Nomenclatural, and Conservation Updates

**DOI:** 10.3390/plants15132092

**Published:** 2026-07-05

**Authors:** Ridha El Mokni, Giulio Barone, Helmut Freitag, Gianniantonio Domina

**Affiliations:** 1 Laboratory of Botany, Cryptogamy and Plant Biology, Faculty of Pharmacy of Monastir, Monastir University, Avenue Avicenna, Monastir 5000, Tunisia; 2 Laboratory of Forest Ecology, National Research Institute of Rural Engineering, Water and Forests, Carthage University, Institution of Agricultural Research and Higher Education (IRESA), Ariana 2080, Tunisia; 3 Department of Agricultural, Food and Forest Sciences, University of Palermo, Viale delle Scienze, Bldg. 4, 90128 Palermo, Italy; gianniantonio.domina@unipa.it; 4 Institute of Biology, University of Kassel, 34109 Kassel, Germany; hfreitag@uni-kassel.de

**Keywords:** conservation, *Gymnospermae*, nomenclature, North Africa, taxonomy

## Abstract

This study provides a revision of the genus *Ephedra* (Ephedraceae) in Tunisia, including taxonomic, nomenclatural, and conservation updates. The genus is represented in Tunisia by four species: *Ephedra altissima*, *E. alenda*, *E*. *fragilis*, and *E*. *nebrodensis*. The first one includes the two varieties *altissima* and *wettsteinii*, while *E. fragilis* is subdivided into the varieties *E. fragilis* var. *fragilis* and *E. fragilis* var. *aurea. E. alenda* was so far usually considered to be a subspecies of *E. alata* but morphological arguments and geographical separation are in favor of species rank. *E. fragilis* subsp. *fragilis* var. *aurea* is recombined here from the recently described *E. aurea*, so far known from Sicily only but reported here for the first time from North Africa. Further studies might show that *E. alata* and *E. procera* also occur in Tunisia. Along with the nomenclatural clarification of the taxa formerly reported from Tunisia including the lectotypification of *E. altissima*, *E. alenda*, and *E. fragilis*, all occurrences of the species in the country were checked and listed. An identification key is also given and supported by pertinent photographs. Conservation concerns are highlighted, particularly for *E. fragilis* var. *aurea*, threatened by habitat destruction due to urbanization and fires, and for the extremely rare *E. nebrodensis*. In a subsequent manuscript, the authors plan to extend the taxonomic studies to the North African *Ephedra* species occurring only outside of Tunisia.

## 1. Introduction

*Ephedra* L. (Ephedraceae, Gymnospermae) is a genus known for its much-branched shrubby to sub-shrubby habit with woody, articulated, flexible slender, horsetail-like stems. It includes ca. 70 species distributed across both the Old and New World [1,2,3,4,5,6,7]. These species are perennial, dioecious, and xeromorphic with assimilating branches, and opposing or whorled leaves which are frequently reduced to more or less membranous sheaths [8]. The reproductive structures are small cones. The male cones (staminate strobili) are arranged in branched inflorescences. They contain 3–12 flowers, each subtended by a bract, consisting of a two-lipped perianth and a staminal column with 2–9 anthers. The female cones (ovulate strobili) are solitary or in groups of 2–3, subtended by 2–4(–6) pairs of bracts and contain 1–3 ovules, each with a more or less pronounced apical tube (micropyle). In fruiting, the bracts either become fleshy and reddish (rarely yellowish to golden-yellow or white) or membranous. The dark colored hard-shelled seeds are angled and more or less lanceolate or ovate in outline. Several *Ephedra* species belong moreover to the oldest medicinal plants known to humankind due to their high contents of ephedrine-type alkaloids in aerial parts and their several pharmacological properties [9,10,11].

According to the more recent surveys, in North Africa (including the Canary Islands), the genus is represented by eight species with several subspecies and varieties [12,13,14,15,16]. Among them according to [17,18,19], only four species are present within the Tunisian territory: *E. altissima* Desf., *E. alata* subsp. *alenda* (Stapf) Trab., *E. fragilis* Desf. subsp. *fragilis*, and *E. nebrodensis* Guss. With respect to phylogenetic relationships, according to ITS sequence studies by [20,21,22], all four species unanimously belong to the basal grade of species distributed in North Africa and the Mediterranean. Based on two samples from Algeria, the latter authors also added *E*. *major* subsp. *procera* to the list, but so far, we remain skeptical about them. Furthermore, during the ongoing floristic studies in the Kroumiria region of northwestern Tunisia (e.g., [23,24,25]), another taxon was detected which turned out to be identical with *E. aurea* that Brullo et al. just recently published from Sicily [26].

The aim of this study was to revise the *Ephedra* taxa occurring in Tunisia in order to check their specific and infra-specific names, align them with the taxonomic consensus in the neighboring Mediterranean countries, provide a full survey about their distribution in Tunisia, assess their conservation status, and designate nomenclatural types. Particular attention was paid to *E. alata* subsp. *alenda* and *E. aurea*, as their taxonomic position was considered uncertain from the beginning of the study.

## 2. Results

**Key to Tunisian taxa of *Ephedra* based on both sexual and vegetative characters**

1. Plant scandent to climbing, stems weak, flexible, requiring support to grow vertically………………………………………………………………..…………………………...… **2**

– Plant erect, stems rigid, able to stand vertically without support……………………………………….........................................................................………. **3**

2. Climbing plant up to 5(10) m tall, leaves up to 1.5 cm long, terminal branches slender, 1–1.5 mm in diameter, pendulous, male cones in richly branched panicles, anthers 2–3 ………………………………………………………………………..……..……… ***E. altissima***

– Scandent up to 2(3) m tall or erect shrub, leaves much shorter, terminal branches robust, more than 1.5 mm in diam., erect or spreading, male cones in simpler inflorescences, anthers 4–6 …….……………………………………..…………….………… ***E. fragilis***

3. Bracts of female cones membranous, broadly scarious-winged, not fleshy, occurring in desert areas …..…………………………………………………………...…... ***E. alenda***

– Bracts of female cones becoming fleshy, colored, never winged, occurring in less arid areas ……………………………………………………..…………………………………. **4**

4. Loosely branched shrub (up to 3 m tall), usually brittle on drying, twigs 2–3 mm in diameter, bracts of female cones usually with ciliolate margin, the innermost connate for 3/4 to 4/5, anthers 4–6, occurring at lower elevations (up to ca. 400 m a.s.l.)…… .. ***E. fragilis***

– Densely branched shrublet or small shrub up to 1 (1.5) m tall, not brittle, twigs 0.7–1 mm in diameter, bracts of female cones not ciliolate, the innermost connate for 1/3 to 1/2, anthers 6–8, occurring at medium to high elevations …………………..…….. ***E. nebrodensis***

**Taxonomic treatment and discussion**

**Figure 1:**
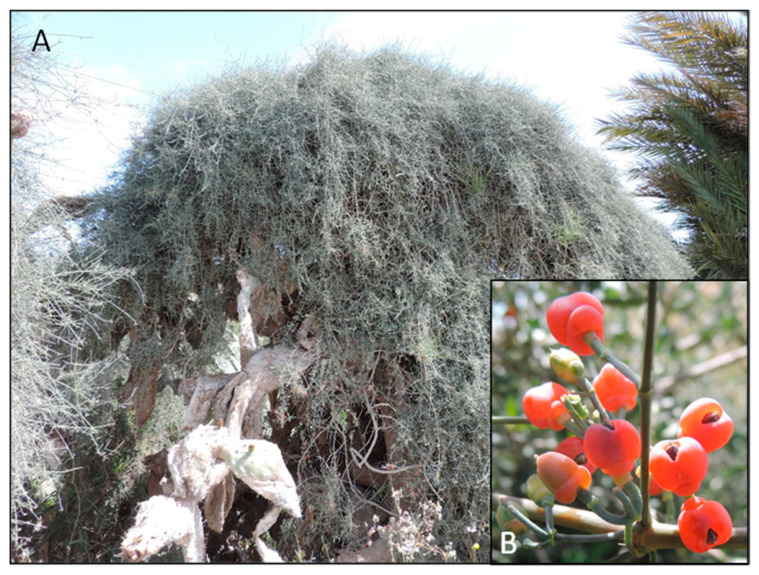
*Ephedra altissima*. (**A**) Habit; (**B**) fruiting strobili (all photos: R. El Mokni, Tunisia, Sousse).

1. ***Ephedra altissima*** Desf. in Méd. Eclairée Sci. Phys. 3(6):163.1792; more elaborate in Desf., Fl. Atlant. 2: 371. 1799. (Figure 1.)

**Type (Lectotype designated here):** *Ephedra altissima*, “Mt. athlas, alger et tunis” FI! (Herb. Webb. 206435); paratype: “de fez, 20 août 1788” MPU013181 photo!

**Nomenclatural notes:** Desfontaines described *E. altissima* from the mountains of the Atlas and published an iconography that clearly represents the diagnostic characteristics [27] (p. 253). We found two original specimens with labels in Desfontaines’s handwriting in FI and in MPU and have designated that from the first place as lectotype because it contains among five separately mounted twigs one with female and three with male organs, while the specimen in MPU just consists of richly branched twigs and one separately mounted male cone with two flowers. Both specimens fit the description and correspond to the current use of the name.

**Taxonomic notes:** The species was subdivided by [8] into the two weakly separated varieties: *algerica* (Atlas Mts. of Algeria and Tunisia) and *mauretanica* (Morocco and W Algeria). These names were overtaken by [12] who added the varieties *scabra* Trabut (Algeria) and *tripolitana* Pamp. (Libya). In Tunisia, except for one specimen from Tatahouine (MPU026465), all collections belong to the first variety, which, however, should be named var. *altissima* because, according to the International Code of Nomenclature [28] (art. 52.1), the name var. *algerica* is nomenclaturally superfluous and therefore illegitimate. The var. *tripolitana* differs only but significantly by an elongated and screw-like micropyle.

**Chorology:** The species has a native range that extends within Canary Islands (Tenerife), N. Africa to Sahara.

**var. *tripolitana*** Pamp. in Bull. Soc. Bot. Ital. 11: Pl. Tripol. 5 (1914).

**Type** (Lectotype designed by Freitag & Maier-Stolte [29] (p. 72)): LIBYA. Tripolitania, Tarhuna: Uadi Sart (2122), 03/04/1913, *R. Pampanini s.n.* (FI006618!).

**Phenology:** Cones produced from January to May and seeds ripening from May to August, depending on altitude.

**Figure 2:**
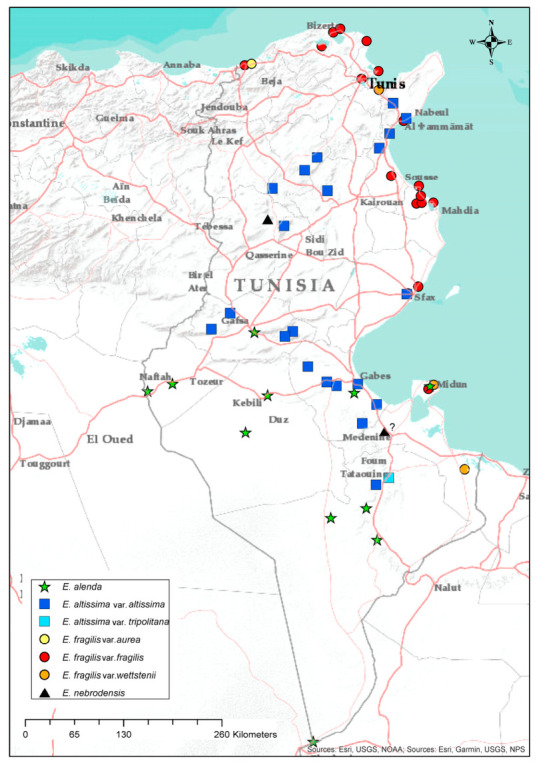
Distribution map of *Ephedra* taxa occurring in Tunisia according to the studied specimens.

**Distribution and habitat** (Figure 2): Throughout the whole country except for the desert regions, from sea level to 1200 m. It grows in more or less degraded Mediterranean shrub communities, preferably in hedges. The var. *tripolitana* seems to be restricted to the coastal areas in NE Tunisia.

**Conservation:** *E. altissima* has an extensive distribution from north to south Tunisia and some of its natural populations occur in the National Parks of Bouhedma [Mezzouna in TC, Governorate Sidi Bouzid & Gov. Gafsa] and of Chaâmbi [TC, Gov. Kasserine]. Its extent of occurrence in Tunisia is 51,429 km^2^ and the area of occupancy is 84 km^2^. Based on the Threats Classification Scheme by IUCN [30], we identified the following impacts: (A) high grazing intensity with numerous goat livestock, which can cause regression of its natural habitats and populations; (B) high number of fires induced during clashes between jihadists and soldiers since 2015, which can cause disruption and fragmentation of natural habitats and populations.

**Additional specimens seen**:

**var. *altissima***: [CB, Gov. Nabeul]: Broussailles du littoral, à Seillonville près d’Hammamet, 8/4/1944, *A. Dubuis s.n.* (MPU329591, P01633272); [TC, Gov. Sousse]: In collibus lapidosis prope La Takrouna (Buficha), 17/1/1886, *A. Letourneux s.n.* (P01582869); [TC, Gov. Sousse]: Environs de Bou Ficha, 30/3/1953, s.c. [manu *G. Pottier-Alapetite s.n.*] (TL0007422); [TC, Gov. Sousse]: Enfidha, Takrouna, within steppic floristic cortege on rocky substrates, 20–30 m a.s.l., 13/11/2021, *R. El Mokni s.n.* (Herb. Univ. Monastir), *ibidem*, 19/11/2022, *R. El Mokni s.n.* (Herb. Univ. Monastir), *ibidem*, 19/11/2024, *R. El Mokni s.n.* (Herb. Univ. Monastir); [DT, Gov. Kairouan]: Djebel Ousselet, village ruiné, 21/3/1953, *G. Pottier-Alapetite s.n.* (MPU026463, MPU026464); [TC, Gov. Sfax]: Sfax, 3/1909, *C.J. Pitard 640* (LY0666190, LY0666195, MARS60603, BP [together with *E. fragilis*]); [DT, Gov. Ben Arous]: Route du Mornag, 1/[19]48, *G. Pottier-Alapetite s.n.* (MPU026462); [DT, Gov. Siliana]: Kef El Rezaï, 24/5/1887, *A. Letourneux s.n.* (P01582865); [TC, Gov. Siliana]: Ad rupes infra Kef Er Rezaï, 24/5/1887, *A. Letourneux s.n.* (P01582921); [TC, Gov. Siliana]: Djebel Bargou, Siliana, 26/5/1903, *S. Murbeck s.n.* (LD1992736), Djebel Serdj, 30/5/1903, *S. Murbeck s.n.* (LD1977994); [DT, Gov. Sidi Bouzid]: Foum El guelta (Djeb. Meghila), 16/5/1887, *A. Letourneux s.n.* (P01582918); [TS, Gov. Gabès]: Ogla Beni Zid, [19th century] s.d., *André s.n.* (P01582898); Gabès, 3/1907, *C.J. Pitard 265* (L0789881, L0789882, L0789883); [TS, Gov. Gabès]: Gabès (Kanzeria), 3/1907, *C.J. Pitard 265* (LY0666231, B101028793, LY0666232, P01582077); [TS, Gov. Gabès]: Gabès (Kanzeria), 3/1907, *C.J. Pitard 266* (LY0666227, B101028789), LY0666234, P01582909, LY0666229); [TS, Gov. Gabès]: Gabès, 3/1909, *C.J. Pitard s.n.* (LY0666196, LY0666207, LY0666208, LY0666210, LY0666212, LY0666213); [TS, Gov. Gabès]: C Sebkhet Zarkin près de Gabès, 4/4/1912, *H. Humbert s.n.* (P06775703); [TS, Gov. Gafsa]: Metlaoui, gorges de Seldja, 3/4/2009, *A. Herrero & al.* (AH3831, AH3893 (MA796732, MA795073, VAL200870, VAL200871, B100478048), ibid. 25/03/2009 (B100478048); [TS, Gov. Gafsa]: Djeb. Attigue, 17/5/1884, *N. Doumet-Adanson & E. Bonnet s.n.* (P01582887); [TS, Gov. Gafsa]: Oued Eddej, 24/4/[1884, *N. Doumet-Adanson & E. Bonnet s.n.* (P01582913a); [TS, Gov. Gafsa]: Dj. Hattig, 17/5/1884, *Doumet s.n.* (P01582913b); [TS, Gov. Medenine]: Djebel Aziza (Djebal Halouga), 26/5/1884, *A. Letourneux s.n.* (P01582886); [TS, Gov. Tataouine]: Djebel Aziza prope El Hamma in faucibus et ad rupes, 26/5/1884, *A. Letourneux s.n.* (P01582885); [TS, Gov. Tataouine]: Dj. Bou Hadid prope Douiret, 7/4/1887, *A. Letourneux s.n.* (P015828669, P01582916, P01582917); [TS, Gov. Gafsa]: Khanguet Mâalla, 11/04/2026, *R. El Mokni s.n.* (Herb. Univ. Monastir); [TS, Gov. Gabès]: Toujène, 12/04/2026, *R. El Mokni s.n.* (Herb. Univ. Monastir).

**var. *tripolitana***: [TS, Gov. Tataouine]: Poste optique de Tatahouine, 31/4/1952, *G. Pottier-Alapetite s.n.* (MPU026465).

**Figure 3:**
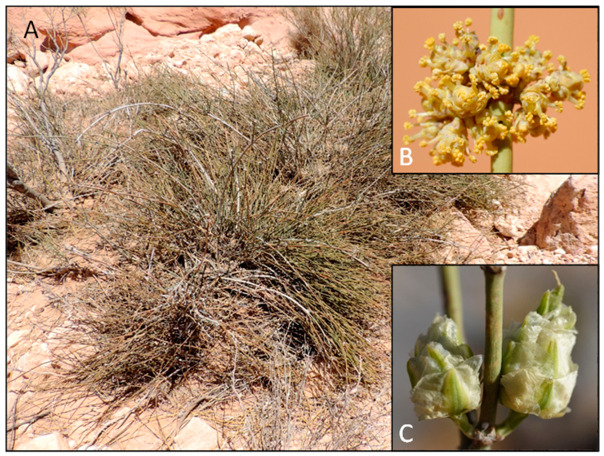
*Ephedra alenda.* (**A**) Habit; (**B**) male strobili; (**C**) female strobili in flower (right) and in bud (left) (photos: A. R. El Mokni, Tunisia, TS; B–C. Lemmel, Morocco, Erg Znaigui).

**2. *Ephedra alenda*** (Stapf) Andr. in Bot. Jahrb. Syst. 64: 262–263. 1931. (Figure 3, Table 1)

Bas.: *Ephedra alata* var. *alenda* Stapf in Denkschr. Kaiserl. Akad. Wiss. Wien, Math.-Naturwiss. Kl. 56(2): 38. 1889.

≡ *Ephedra alata* subsp. *alenda* (Stapf) Trab. in Battandier & Trabut, Fl. Algérie Tunisie: 399. 1905.

**Type (Lectotype designated here):** L. Kralik Plantae Algeriensis selectae 1858, 85. *Ephedra alata* ♀ Decne. Florul. Sinaic. in Ann. Sc. nat. sér 2, II, 239, Arabice Alenda. In aggeribus arenae mobilis, loco, a copia plantae, Oued el Alenda dicto, inter urbem el Oued et montem arenosum, Djebel Kref, in ditione Souf, 20.IV.1858 (MPU027025!; iso: BP!, FI!, GOET!, MPU027026!, P01634360!, P01634367!, P01634398!).

**Nomenclatural notes:** The protologue reports many specimens from Morocco, Algeria, and Tunisia. Here, we select the specimen MPU027025 as the lectotype of the name. It includes two segments of branches of a female individual, fits the original description, and has duplicates in some other herbaria (see the forgoing paragraph). The specimen corresponds to the current use of the name.

**Taxonomic notes:** Whereas in almost all treatments, this taxon has been regarded as a subspecies of *E. alata*, due to the high number of differential characteristics (see Table 1) here, we accept the specific rank already proposed by [31], but followed so far only by [32] without respective discussion. The most striking differences which are not bridged by intermediates refer to some obvious strobili characteristics. Habit (size) is less reliable, as the potential size is often not reached in younger individuals, under frequent browsing pressures, or in unfavorable habitats.

Only from the most recent observations by [33] in Morocco and from the authors’ own observations in the Tataouine governorate of Tunisia do we know that *E. alenda* also grows on stony and rocky ground where the bushes remain smaller. So far, the species was usually considered to be a typical psammophyte in contrast to *E. alata* [12,31].

**Chorology:** The species has a restricted native range limited to N. Africa (including Algeria, Egypt, Libya, Mauritania, Morocco, Tunisia) with Western Sahara.

**Phenology:** Cones produced in March–April.

**Distribution and habitat in Tunisia** (Figure 2)**:** South Tunisia. Saharan sands, but in south Algeria and Morocco, it also occurs on stony and rocky habitats, particularly in the Tataouine governorate.

**Conservation:** All populations currently known occur in the southern part of Tunisia, from the National Parks of Dghoumès (Tozeur) and Senghar-Jabbes (Remada, Gov. Tatouine); others (male individuals only) are recently reported from Djerba [TS, Gov. Medenine]. The extent of occurrence in Tunisia is 70,824 km^2^ and the area of occupancy is 44 km^2^. Global changes can negatively affect habitats and populations.

**Additional specimens seen**: [TS, Gov. Gafsa]: Maguebra (Oued Mlik), 8/4/1858, *E. Cosson s.n.* (P01636326); [TS, Gov. Kebili]: Bir El Ghabi, près d’chott, 14/3/1875, *Duveyrier. 1016* (P016344359); [TS, Gov. Tozeur]: Nefta, 4/1889, *P. Cellier s.n.* (P01655621); [TS, Gov. Tozeur]: Pl. de Nefta, 11/6/1927, *C. Dumont s.n.* (BC879363, BC879364, MPU981059, P06775693, P06775694); [TS, Gov. Kebili]: Nefzaoua mérid., 18/3/1887, *A. Letourneux s.n.* (P01655612); [TS, Gov. Kebili]: Inter Bir el Hachechina et Sabria, 17/3/1887, *A. Letourneux s.n.* (P01655610, P01655611, P01655613, UPS); [TS, Gov. Kebili]: Bir El-Arefdji *A. Letourneux* 21/4/1887 (P01582042, FI); [TS, Gov. Kebili]: Bir el Asli, *A. Letourneux* 29/4/1887 (P01582043); [TS, Gov. Tataouine]: Près de Fort Saint, 15/5/1951, *G. Pottier-Alapetite s.n*. (MPU026461); [TS, Gov. Tataouine]: Kambout, within steppic floristic cortege on edges of streams, 410–420 m a.s.l., 15/05/2021, *R. El Mokni s.n.* (Herb. Univ. Monastir); [TS, Gov. Medenine]: Djerba, sur la route de Midoun vers Houmet Souk; Ghizen, 4/04/2026, *R. El Mokni s.n.* (Herb. Univ. Monastir); [TS, Gov. Gabès]: sur la route de M’dou vers Matmata, 12/04/2026, *R. El Mokni s.n.* (Herb. Univ. Monastir).

**Figure 4:**
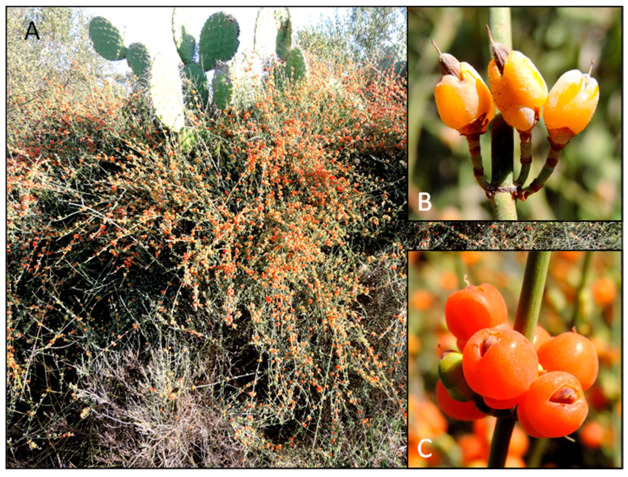
*Ephedra fragilis.* (**A**) Habit; (**B**) fruiting strobili of var. *aurea*; (**C**) fruiting strobili of var. *wettsteinii* (all photos: R. El Mokni, Tunisia, TC & K).

**3. *Ephedra fragilis*** Desf., Fl. Atlant. 2: 372. 1799. (Figure 4.)

**Type (Lectotype designated here):** (ALGERIA) “in montibus ad maris litora” 1774? [manu Desfontaines] P-Desf. P0700328!, para: FI 069484!

= *E. gibraltarica* Boiss., Fl. Orient. 5/2: 714. 1884.

**Nomenclatural notes:** The protologue reports: “Habitat in montibus ad maris litora” [Habitat in the mountains near the seashore]. We detected two original specimens: a male in P-Desf., made up of six separately mounted flowering branches associated with a hand-written draft of the diagnosis, and a female in FI from the *Herbarium Webbianum* with the only remark “ex herb. Desf.”. Though it was tempting to designate the female specimen as the lectotype, we decided in favor of the male and consider the female as a paratype because it bears a single female cone only, which is actually separated from the plant. It was recognized as such only after careful removal from overlaying branches and preparation by the curator of FI (Chiara Nepi). In addition,, with the long exerting anther column and the head-like complex of sessile anthers, the male plants differ more strikingly from related species (e.g., from *E. alata*). According to [8: 54], Desfontaines did the collection 1774 W of Alger and from the littoral of the Bay of Alger. The lecto- and the paratype fit the description and correspond to the current use of the name.

**Taxonomic notes:** The extraordinary polymorphism of the species was recorded by [8] but he separated only var. *desfontainii* and var. *campylopoda.* While today, the first name has to be replaced by var. *fragilis* as it includes the type of the species [28] (art. 52.1), the second variety, which is restricted to the Central and Eastern Mediterranean, is generally considered as the separate species *E. foeminea* Forssk. Buxbaum [34] described *E. wettsteinii* based on its long and contorted micropyle but stressed its close affinity to *E. fragilis.* Maire [12] raised var. *desfontainii* and Stapf’s subvar. *cossonii,* from the dry interior mountains of the Atlas system, to subspecies level. At the same time, he reduced *E. wettsteinii* to a variety of the first, alongside var. *dissoluta,* based on the subvariety *dissoluta* Stapf, and var. *gibraltarica* based on *E. gibraltarica* Boiss. Finally, [26] described *E. aurea* from Sicily—but recently we also detected it on the NW Tunisian coast—which differs from typical *E. fragilis* by yellow to orange-colored fruiting cones (see below).

In Tunisia, we recognize only the following infraspecific taxa:

**var. *fragilis*** the most common form (Figure 4A,C).

**var. *wettsteinii*** (Buxb.) Maire & Weiller in Maire, Contrib. 1939: 2879.

**Type (Lectotype designated here):** TUNISIA, “auf den Wällen der Gärten westlich der Stadt Sfax (Buxb.et Schussnig). “Sfax, an der Strasse nach El Djem”, 26/4/1924, *F. Buxbaum & B. Schussnig s.n.* (WU0062057!; iso: WU0062058!, B100166433!, GZU000267223!, K000076234!, WU0062067! WU0062057!, WU0062057!, GAZ000267223.

≡ *E. wettsteinii* Buxb. in Verh. Zool.-Bot. Ges. Wien 76: 36. 1927.

**Taxonomic Notes**: The micropyle is said in the protologue to have a total length of about 4–5 mm and is contorted. However, our measurements carried out on more than 30 specimens, including the rich type material, show an overall length (direct distance) of the sometimes only slightly contorted tube of about 1.5–3 mm only. Further characters given in the description are weak, e.g., instead of 2–4 anthers, sometimes we counted 4–6, and particularly dense female inflorescences also occur sometimes in otherwise typical individuals of *E. fragilis*. As the authors also state that in habit and in vegetative characters, their species fully agrees with *E. fragilis* and furthermore grows in the same ecological niche, the varietal rank appears to be justified though it was denied by [35]. Shape and length of the micropyle have some taxonomic significance (see also var. *tripolitana* in *E. altissima*) that also led to the separation of subspecies *helvetica* (C.A. Meyer) Aschers. & Graebn.in *E. distachya* L. and even the definition of species as in *E. intermedia* Schrenk & C.A.Mey.

**Chorology:** The species has an extended native range within Macaronesia, W. & Central Medit (including Algeria, Baleares, Canary Is., Italy, Libya, Madeira, Morocco, Portugal, Sicilia, Spain, Tunisia, Western Sahara).

**var. *aurea*** (Brullo et al.) El Mokni, Barone, Freitag & Domina **comb. nov.** (Figure 4B)

Type: ITALY, Sicily: Trapani, Penisola di San Vito lo Capo, versante costiero occidentale in località “Isulidda” a Nord di Macari, 50 m di altitudine, 38°08′44″ N, 12°44′09″ E, 30 June 2012, S. Brullo & V. Ilardi s.n. (holotype, CAT; isotypes, CAT).

≡ *E. aurea* Brullo et al. in Phytotaxa: 530: 9. 2020.

**Taxonomic Notes:** *Ephedra aurea* is here downgraded to varietal rank because it differs from the nearby growing typical *E. fragilis* only by the yellow to orange color of the bracts in mature female cones. However, such varieties which are caused by the partial deficiency of carotenoid synthesis also occur in several other *Ephedra* species such as *E. procera* in the Caucasus and N Turkey, *E. distachya* in the Caucasus, and *E. intermedia* in the western Himalayas. They are reported also from *E. nebrodensis* in the Moroccan part of the Atlas Mts. and were classified by Maire [12] (p. 162) just as *E. chrysocarpa.* Inexplicably, the authors compared their new species only with *E. nebrodensis* while in its much more robust habit, in the structure of female cones (with upper bracts fused for at least 3/4), morphology of leaves (wider than long), pollen morphology (Type A—with more than 10 plicae and unbranched pseudosulci), and furthermore in its ecology (thermo-mediterranean), it agrees with *E. fragilis.*

**Phenology:** Cone production from March to May and ripening of seeds from June to July/August.

**Distribution and habitat** (Figure 2)**:** The species is known so far from Western and Central Mediterranean countries up to the Cyrenaica [32]. In Tunisia, *E. fragilis* var. *fragilis* and *E. fragilis* var. *wettsteinii* are common in more or less degraded shrublands throughout the coastal belt, while var. *fragilis* and var. *aurea* also extend to open plant communities or even woodlands on stony and rocky hillsides.

**Conservation:** According to the IUCN Classification Scheme [30], threats for *E. fragilis* are represented by: (A) the increase in urban areas and roads [which can cause disruption and fragmentation of natural habitats and populations]; (B) the destruction of hedges by planting *Opuntia ficus-indica* (L.) Mill., and being highly infested during the last five years by the introduced Hemiptera *Dactylopius opuntiae*. These hedges remain the best habitat refuge for *E. fragilis* var. *fragilis* and var. *wettsteinii*, but locally, they have been totally eradicated in order to prevent the insect from spreading to other areas. The newly discovered populations of the var. *aurea* in Tunisia occur in two localities within the kermes oak forests of the northwestern part of the country, where only a few dozen mature individuals were recorded within an area of approximately 3 ha. The extent of occurrence in Tunisia of *E. fragilis* is 39,942 km^2^ and the area of occupancy is 84 km^2^. Its habitats are under high risk of fire, excessive grazing and clearing, and extensive deforestation following the expansion of new residences and hotels near the cities and on the costal dunes of Tabarka. More field surveys are needed in the coastal dunes of the northeastern part (including Cap Bon region) of the country within the kermes oak forests to trace the distribution of this taxon along the southern Mediterranean shores.

**Additional specimens seen**:

**var. *fragilis***: [K, Gov. Jendouba]: Dunes de Tabarka, 4/5/1969, *J. Timbal s.n.* (MPU301926).

Tabarka, within shrubby floristic communities of *Quercus coccifera*, 19/5/2024, *R. El Mokni s.n.* (Herb. Univ. Monastir); [TS, Gov. Medenine]: Djerba, 4/1886 L*etourneux s.n.* (P-Coss.); [NE, Gov. Bizerta]: Bizerte, 1/6/1888, *E. Cosson*, *G. Barratte, C. Duval s.n.* (P01582119, P01582122, P01582129, P01655789, P-Coss. K*,*); [NE, Gov. Bizerta]: Djebel Ichkeul, 27/6/1889, *A. Letourneux s.n.* (P01582123, P01582144); [NE, Gov. Bizerta]: Porto Farina, ravin descendant du Kef er Rahma à l’W du village, 27/4/1957, *J. Raynal 1817* (P01582816); c. 5 km SW of Bizerte, sand coast N Habib Arifa, 0–5 m s.no., 37°15′16″ N/9°55′20″ E, 31/3/2014, *N. Ardenghi & al. 1004* (B100708252, MA-01-00909154, PAL-Gr62057); [NE, Gov. Bizerta]: Jebel Ichkeul, S of Lake Ichkeul, c. 8 km W of Menzel Bourgiba, península N and hill W of the mineral well, 29/4/2014, *N. Ardenghi & al. 728* (MA01-00908942, PAL-Gr61781); [NE, Gov. Bizerta]: Bizerta-city, corniche, within shrubby floristic communities of planted pines, 11/7/2022, *R. El Mokni s.n.* (Herb. Univ. Monastir); [NE, Gov. Tunis]: Tunis, Ksar Said, 11/5/1893, *T. Delacour s.n.* (P01621664); [NE, Gov. Tunis]: Environs de Tunis, 8/1880, *A. Roux s.n.* (MPU277213); [TC, Gov. Tunis]: Sfax, in incultis, 2/6/1894, *Kralik* (P-Coss); [TC, Gov. Sfax]: Sfax, in aridis, 3/1909, *Pitard,* Plantes de Tunesie /P-Coss.), together with *E. altissiima*; [TC, Gov. Tunis]: In arenosis maritimis prope La Marsa, 10/4/1938, *R. Maire & M. Weiller s.n.* (MPU277210); [CB, Gov. Nabeul]: Nord d’Hammamet-Sousa, 7/6/1883, *E. Cosson s.n.* (P01582915); [CB, Gov. Nabeul]: Hamamet, in Opuntiis, 22/10/1907 *M. Gandoger 170*, K; [CB, Gov. Nabeul]: *ibidem*, Hamamet-Süd, häufig an Feldmauern, *C. Simon* s. no., s. dat. BAS); [TC, Gov. Sousse]: Div. Sousse, 3–4 km W of Kalaa Kébira, valley with macchia and *Olea* plantation, 7/4/1972, *Bot. Dept. Univ. Stockholm s.n.* (NY00335778, S-C7687); [TC, Gov. Monastir]: Monastir, Jemmal, Ridène, within olive grove hedges, 17/3/2022, *R. El Mokni s.n.* (Herb. Univ. Monastir); [TC, Gov. Monastir]: Touza, within olive grove hedges, 13/5/2022, *R. El Mokni s.n.* (Herb. Univ. Monastir); [TC, Gov. Monastir]: Monastir-city, Falaise, within plantations of *Acacia* sp. pl., 10/4/2023, *R. El Mokni s.n.* (Herb. Univ. Monastir); [TC, Gov. Monastir]: Ksibet Mediouni, within olive grove hedges, 11/6/2023, ibidem, 7/7/2023, *R. El Mokni s.n.* (Herb. Univ. Monastir), ibidem, 27/5/2024, *R. El Mokni s.n.* (Herb. Univ. Monastir); [TC, Gov. Monastir]: Bekalta, within olive grove hedges, 21/5/2024, *R. El Mokni s.n.* (Herb. Univ. Monastir);

**var. *wettsteinii***: [TC, Gov. Sfax]: Sfax, 5/1938, *R. Maire & M. Weiller 1662* (P01655786, MPU045850); WU0062058; In aggeribus et muris hortorum, circa urbem Sfax, 6/5/1938, *R. Maire & M. Weiller s.n.* (MPU277264, MPU277265, P277265); [DT, Gov. Ben Arous]: Djebel Bou Kornine, 15/4/1912, *H. Humbert s.n.* (MPU277212); [DT, Gov. Ben Arous]: Bou Kornine, 15/4/1952, *R. Vigruer s.n.* (P06775689); [DT, Gov. Ben Arous]: Djebel Bou-Kournin, s.d., *J.A. Battandier s.n.* (MPU277244); [TS, Gov. Medenine]: 20 Km SE ad oppido Ben Gardan, 3/5/1938, *R. Maire & M. Weiller s.n.* (MPU277211); [TS, Gov. Medenine]: Medenine, near hotel-zone between Ras Tougeness and Homt souk, 30/5/1997, *C.C. Townsend s.n.* (K000459279).

**var. *aurea***: [K, Gov. Jendouba]: (Kroumirian forests), Tabarka, within floristic cortege of kermes oak forests (sometimes with planted *Pinus* spp., and *Acacia* spp.) on rocky substrates covered with dune sand, 20–30 m a.s.l., 0/07/2024, *R. El Mokni s.n.* (Herb. Univ. Monastir), *ibidem*, 30–40 m a.s.l., 8/8/2024, *R. El Mokni s.n.* (Herb. Univ. Monastir), *ibidem*, 26/8/2025, *R. El Mokni s.n.* (Herb. Univ. Monastir).

**Figure 5:**
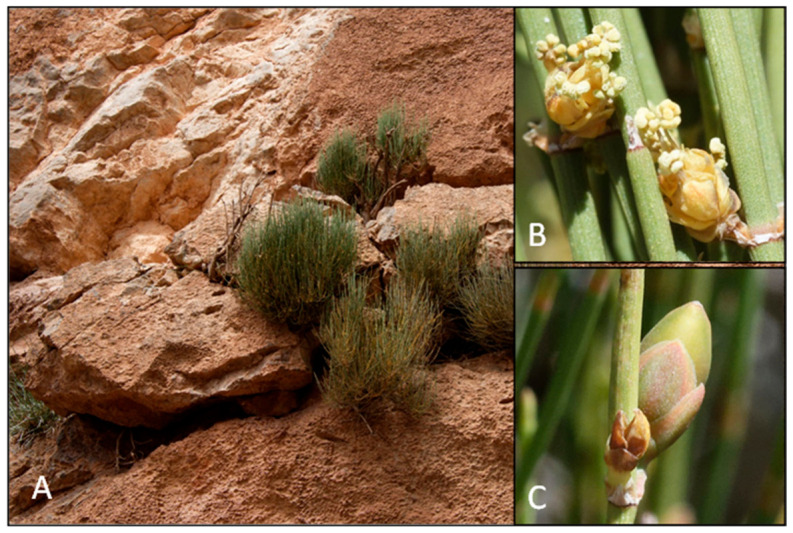
*Ephedra nebrodensis.* (**A**) Habit; (**B**) male strobili; (**C**) fruiting strobilus (all photos: C. Lemmel, Morocco, High Atlas, reproduced with the author’s permission).

**4. *Ephedra nebrodensis*** Tineo ex Guss. Fl. Sicul. Syn. 2: 638. 1844. (Figure 5)

**Type** (Lectotype designated by [36] (p. 91))**:** [ITALY. Sicily], [Madonie, Isnello], 1832, *[V.] Tineo s. n.* (NAP!, Collection “Gussone—Sicilia”).

= *E. major* var. *nebrodensis* (Tineo ex Guss.) Hayek, Repert. Spec. Nov. Regni Veg. Beih. 30(1): 44 (1924).

*= E. villarsii* Gren. & Godr., Fl. France [Grenier] 3: 161 (1855).

= *E. major* subsp. *villarsii* (Gren. & Godr.) P. Fourn. Quatre Fl. France 19 (1934).

**Taxonomic notes:** The name *E. major* Host was rejected following the proposal by Brullo and Del Guacchio [37]. The Tunisian populations require further investigation. To date, all authors except [20,21,22] have recognized only a single species of this group in North Africa, which they called *E. nebrodensis* or *E. major* var. *nebrodensis*. However, ITS sequence data from two samples collected in neighboring Algeria indicate an affinity with the morphologically similar but rather distantly related *E. procera,* a species distributed mainly from the eastern Mediterranean to the Near and Middle East. Even the molecular data obtained from [22] (sub *E. major* from Croatia, close to the sea) do not help to clarify the issue. No molecular data are currently available for *E. nebrodensis* from Sicily or Tunisia, and the available Tunisian material is scanty and does not bear female cones, preventing a reliable assessment of its identity. Attempts to recollect the species were unsuccessful because access to the relevant areas is strictly prohibited. Moreover, the two available Tunisian specimens identified as *E. nebrodensis* were collected at unusually low elevations (c. 600 m) in S-Tunisia, whereas in Sicily and other parts of the Atlas Mountains, the species is generally restricted to higher elevations of at least 1500 m. We therefore hypothesize that both species may occur in North Africa, including Tunisia.

**Chorology:** The species is native to the Canary Islands, Mediterranean to Mauritania (including Albania, Algeria, Cyprus, France, Greece, Italy, Lebanon-Syria, Mauritania, Morocco, NW. Balkan Peninsula, Sardegna, Sicilia, Spain, Tunisia, Turkey).

**Phenology:** Cone production from May to June and ripening of seeds from August to September.

**Distribution and habitat** (Figure 2)**:** Very rare and restricted to limestone cliffs of the highest mountains of the “Dorsale Tunisienne” in Central Tunisia and on the Dahar Mts. in southern Tunisia. The species seems to be undercollected, and the population in southern Tunisia is in need of further studies because the only specimen from there does not bear cones and was collected from unusually low altitudes. Nevertheless, the specimens were already identified by [8] as *E. nebrodensis*. Unfortunately, the access to the respective area is actually prohibited.

**Conservation:** The primary threats are (A) overgrazing by large numbers of goats, leading to habitat and population regression; and (B) frequent fires caused by jihadist–soldier clashes since 2015, resulting in habitat and population disruption and fragmentation. The area of occupancy in Tunisia, based on 2 cells of 2 km of width, corresponds to 8 km^2^.

**Specimens seen**: [SE, Gov. Medenine] Djebel Tadjara (280 m), 6/5/1884, *A. Letourneux s.n*. (P01655677!) (*incompl., det. dub.*); [CE, Gov. Kasserine] Djebel Teioucheha ad rupes fissuris parvula cacumine, 22/5/1887, *A. Letourneux* s.n. (P01655640!, P01655637!, P01655677!).

## 3. Materials and Methods

The present study is based on the morphological examination of both living material from almost all over the country and herbarium specimens. The search for the respective locations started from the data given in the literature [17,38]. A portion of the herbarium specimens was collected by the first author and is housed in his personal herbarium and in the herbarium of the Faculty of Pharmacy of Monastir (here indicated as Herb. Univ. Monastir); many specimens have duplicates in SAF. Numerous specimens from other herbaria were also consulted, either through online resources, including GBIF [39], or by visiting the relevant herbaria or requesting loans: ALF, B, BP, FI, G, GAP, GOET, K, LY, MA, MARS, MPU, NY, P, PAL, TL, WAG, W, WU (codes according to [40]).

In dealing with nomenclatural types, a precautionary approach was adopted, and specimens were designated as lectotypes rather than treated as holotypes, even in cases where only a single locality was cited in the protologue. This approach was adopted because it cannot be established with certainty whether the author had only a single collection available when describing the new taxon. The same practice is now widely followed for most names based on nineteenth-century collections from North Africa [29,41,42].

Identification of *Ephedra* samples by classical morphological characteristics is usually hampered by the fact that the material does not contain male or female organs and that the vegetative branches of the different species only show comparatively few distinguishing characteristics, which might be somewhat variable and/or overlapping. However, that can be compensated for by experience and by observations in the field concerning the habit, though that is very often highly influenced by grazing or cutting, and by ecological parameters, in particular altitude, amount of rainfall, and soil conditions. The most important morphological characteristics used in our study were habit, diameter of green twigs, surface of twigs, color and structure of pith, size and structure of leaves, details of male and female cones, and shape of pollen grains. Detailed descriptions of the taxa are not given because the distinctive characteristics are included in the key in the taxonomic notes and can be retrieved also from the attached photographs. The nomenclature of the associated taxa is according to Euro + Med PlantBase [14] and the African Plant Database [15]. Chorology follows POWO [16].

The following are reported in the taxonomic treatments: synonyms, the localities indicated in the protologues, types, nomenclatural and taxonomic notes, phenology in Tunisia, distribution and habitat in Tunisia, regional conservation notes, and lists of the specimens seen. In them, the arrangement follows the division of Tunisia into the macro-geographical areas by [17].

For each species, the extent of occurrence (EOO) and the area of occupancy (AOO) were calculated using the GeoCAT Tool ver. beta [43]. However, the IUCN regional risk category [30] to be assigned to these species remains DD due to the lack of data on population trends and evidence of the existence of historically known populations.

## Figures and Tables

**Table 1:** Differential characters, habitat, and distribution of *Ephedra alenda* and *E. alata*.

Character	*E. alenda*	*E. alata*
**Size (full grown)**	0.5–2.0 (3.0) m	0.5–1.0 m
**Annual branches**		
Diameter in mm	1.0–1.8 (2.0), delicate	2–3, robust
Surface	clearly sticky	slightly sticky
**Male flowers**		
Anthers	6–8, sessile, headlike united	4–6, distinctly stipitate
Staminal column	clearly exserted from perianth	not exserted from perianth
**Mature female strobili**		
Diameter in mm	10–15	8–9
**Bracts**		
Size in mm	7.5–8.0 × 8.0–8.5	5.5–6.0 × 5.5–6.0
Shape	regularly circular	circular, with stipitate base
**Seeds**		
Length in mm	8–10 (14)	6.0–6.5
Apex	often with 3 prominent denticles	without denticles
**Habitat**	sand deserts, preferably	stony and rocky deserts, preferably
**Distribution**	Morocco to Libya	Libya to S Iraq and Yemen, outpost in S Algeria

## Data Availability

The original contributions presented in this study are included in the article. Further inquiries can be directed to the corresponding authors.

## References

[1] Ickert-Bond S.M. (2003). Systematics of New World Ephedra L. (Ephedraceae): Integrating Morphological and Molecular Data.

[2] Ickert-Bond S.M., Wojciechowski M.F. (2004). Phylogenetic relationships in *Ephedra* (Gnetales): Evidence from nuclear and chloroplast DNA sequence data. Syst. Bot..

[3] Huang J., Giannasi D.E., Price R.A. (2005). Phylogenetic relationships in *Ephedra* (Ephedraceae) inferred from chloroplast and nuclear DNA sequences. Mol. Phylogenet. Evol..

[4] Ickert-Bond S.M., Rydin C., Renner S.S. (2009). A fossil-calibrated relaxed clock for *Ephedra* indicates an Oligocene age for the divergence of Asian and New World clades and Miocene dispersal into South America. J. Syst. Evol..

[5] Hollander J.L., Vander Wall S.B., Baguley J.G. (2010). Evolution of seed dispersal in North American *Ephedra*. Evol. Ecol..

[6] Ickert-Bond S.M., Renner S.S. (2016). The Gnetales: Recent insights on their morphology, reproductive biology, chromosome numbers, biogeography, and divergence times. J. Syst. Evol..

[7] Christenhusz M., Byng J. (2016). The number of known plant species in the World and its annual in-crease. Phytotaxa.

[8] Stapf O. (1889). Die Arten der Gattung Ephedra. Denkschriften der Kaiserlichen Akademie der Wissenschaften, Mathematisch-Naturwissenschaftliche Klasse, Akademie der Wissenschaften. Wien Mathematisch-Naturwissenschaftliche Klasse.

[9] Al-Snafi A.E. (2017). Therapeutic importance of *Ephedra alata* and *Ephedra foliata*, A review. Indo Am. J. Pharm. Sci..

[10] González-Juárez D.E., Escobedo-Moratilla A., Flores J., Hidalgo-Figueroa S., Martínez-Tagüeña N., Morales-Jiménez J., Muñiz-Ramírez A., Pastor-Palacios G., Pérez-Miranda S., Ramírez-Hernández A. (2020). A Review of the *Ephedra* genus: Distribution, Ecology, Ethnobotany, Phytochemistry and Pharmacological Properties. Molecules.

[11] Hadjadj K., Daoudi B.B., Guerine L. (2020). Importance thérapeutique de la plante *Ephedra alata* subsp. *alenda* dans la médecine traditionnelle pour la population de la région de Guettara (Djelfa, Algérie). Lejeunia.

[12] Maire R. (1952). Flore de l’Afrique du Nord.

[13] Dobignard A., Chatelain C. (2011). Index synonymique de la flore d’Afrique du Nord. Vol.1: Pteridophyta, Gymnospermae, Monocotyledoneae.

[14] Raab-Straube E. von 2022+ Ephedraceae. In Euro+Med PlantBase—The Information Resource for Euro-Mediterranean Plant Diversity. https://europlusmed.org/cdm_dataportal/taxon/7c36779e-31f6-45be-baca-f6be4772e982.

[15] APD [African Plant Database] African Plant Database (Version 3.4.0). *Ephedra* L.. https://africanplantdatabase.ch/en/nomen/genus/190540/ephedra-l.

[16] POWO [Plants of the World Online] *Ephedra* Tourn. ex L. Royal Botanic Gardens, Kew. https://powo.science.kew.org/taxon/urn:lsid:ipni.org:names:383433-1.

[17] Cuénod A., Pottier-Alapetite G., Labbe A. (1954). *Ephedra* L.. Flore Analytique et Synoptique de la Tunisie: Cryptogames Vasculaires, Gymnospermes et Monocotylédones.

[18] Greuter W., Burdet H.M., Long G. (1984). MED-CHECKLIST: A Critical Inventory of Vascular Plants of the Circum-Mediterranean Countries—Volume 1 (Pteridophyta).

[19] Daoud-Bouattour A., Muller S.D., Ghrabi-Gammar Z., Ben Haj Jilani I., Le Floc’h E. (2025). Flore de Tunisie, Index Synonymique Commenté.

[20] Rydin C., Korall P. (2009). Evolutionary relationships in Ephedra (Gnetales)—With implications for seed plant phylogeny. Int. J. Plant Sci..

[21] Rydin C., Khodabandeh A., Endress P.K. (2010). The female reproductive unit of *Ephedra* (Gnetales): Comparative morphology and evolutionary perspectives. Bot. J. Linn. Soc..

[22] Rydin C., Blokzijl R., Thureborn O., Wikström N. (2021). Node ages, relationships, and phylogenomic incongruence in an ancient gymnosperm lineage—Phylogeny of Ephedra revisited. Taxon.

[23] El Mokni R. (2018). *Serapias* × *debelairii*, a new natural hybrid from Tunisia within a sympatric population of *S. stenopetala* and *S. parviflora*. J. Eur. Orch..

[24] El Mokni R. (2020). *Echinophora spinosa* L. (Apiaceae), a new species in the flora of Tunisia and second report from North Africa. Hacquetia.

[25] El Mokni R. (2024). New records of coniferous species (Gymnospermae, Pinidae) for the woody flora of Tunisia and North Africa. Hacquetia.

[26] Brullo S., Brullo C., Cambria S., Ilardi V., Siracusa G., Giusso del Galdo G. (2022). *Ephedra aurea* (Ephedraceae), a new species from Sicily. Phytotaxa.

[27] Desfontaines R.L. (1799). Flora Atlantica: Sive Historia Plantarum Quae in Atlante, agro Tunetano et Algeriensi Crescunt.

[28] Editorial Committee of the Madrid Code (2025). International Code of Nomenclature for Algae, Fungi, and Plants (Madrid Code).

[29] Freitag H., Maier-Stolte M., Cuccuini P., Nepi C., Abuhadra M.N., Cecchi L., Freitag H., Luccioli E., Maier-Stolte M., Marcucci R., Peruzzi L., Pignotti L. (2015). Ephedraceae. The Libyan Collections in FI (Herbarium Centrale Italicum and Webb Herbarium) and Studies on the Libyan Flora by R. Pampanini—Part 1.

[30] IUCN [International Union for Conservation of Nature] The IUCN Red List of Threatened Species. 2025-2. Version 3.3. https://www.iucnredlist.org/resources/threat-classifi-scheme.

[31] Andreánsky G. (1931). Beiträge zur Kenntnis der nordafrikanischen Arten der Gattung *Ephedra*. Bot. Jahrb. Syst..

[32] Freitag H., Maier-Stolte M., Browicz K. (1994). Ephedraceae. Chorology of Trees and Shrubs in South-West Asia and Adjacent Regions.

[33] Lemmel C. Flore saharienne du Maghreb, Vers. 2.1. http://atlas-sahara.org.

[34] Buxbaum F. (1927). Beitrag zur Flora von Tunesien. Verh. Zool.-Bot. Ges. Wien.

[35] Le Floc’h E., Boulos L., Véla E. (2010). Catalogue Synonymique Commenté de la flore de Tunisie.

[36] Del Guacchio E., Cambria S., Brullo S. (2021). Typification of the name *Ephedra nebrodensis* (Ephedraceae). Phytotaxa.

[37] Brullo S., Del Guacchio E. (2021). Proposal to reject the name *Ephedra major* (Ephedraceae). Taxon.

[38] Quézel P., Santa S. (1963). The genus *Ephedra* L.. Nouvelle Flore d’Algérie et des Régions Désertiques Méridionales.

[39] GBIF.org, GBIF Home Page. https://www.gbif.org.

[40] Thiers B. Index Herbariorum: A Global Directory of Public Herbaria and Associated Staff. New York Botanical Garden’s Virtual Herbarium. http://sweetgum.nybg.org/ih.

[41] Domina G., Greuter W., Marino P., Schäfer P.A. (2013). Types of names of *Orobanche* taxa described from North Africa. Plant Biosyst..

[42] Villar J.L., Alonso M.Á., Crespo M.B., Martínez-Azorín M. (2023). Nomenclatural Type Identification of Names in North African *Tamarix* (*Tamaricaceae*). Plants.

[43] Bachman S., Moat J., Hill A.W., de la Torre J., Scott B. (2011). Supporting Red List threat assessments with GeoCAT: Geospatial conservation assessment tool. ZooKeys.

